# 

**Published:** 1894-10

**Authors:** 


### CONTENTS.

ORIGINAL COMMUNICATIONS—

The Treatment of Puerperal Peritonitis. By Virgil O. Hardon, M.D., Atlanta, Ga., Professor of Obstetrics and Gynecology in the Atlanta Medical College..... 449

Tetanus. By Charles J. Montgomery, M.D., Augusta, Ga............ 45

The History of Medicine and Surgery in Georgia. By L. B. Grandy, M.D., Atlanta, Ga............ 459

Clinical Lecture on Psoriasis. By William S. Gottheil, M.D., Dermatologist to the Lebanon Hospital, the Northwestern and the German West-Side Dispensaries, New York............ 466

SOCIETY REPORTS—

Augusta Academy of Medicine............ 470

Atlanta Obstetrical Society............ 474

CORRESPONDENCE—

Medical News from New York............ 477

EDITORIAL—

Proposed Bill for Three Boards of Examiners............ 481

Legislation for the Prevention of Blindness............ 484

The Tri-State Medical Society............ 486

SELECTIONS AND ABSTRACTS—

Eye, Ear, Nose, and Throat............ 489

Skin Diseases and Syphilis............ 492

Surgery............ 498

MEDICAL ITEMS............ 509

BOOK REVIEWS............ 511

ADVERTISERS' INDEX TO ADVERTISEMENTS............ xii

CLASSIFIED INDEX TO ADVERTISEMENTS............ xxxv

ITEMS OF INTEREST............ x, xii

